# Effect of intravitreal VEGF inhibitors on renal-related adverse events in patients with diabetes mellitus: systematic review and meta-analysis

**DOI:** 10.3389/fphar.2025.1691597

**Published:** 2025-11-14

**Authors:** Yu Zheng, Fan Zhang, Xueling Li, Xianwen Zhang, Yifei Zhong

**Affiliations:** Department of Nephrology, Longhua Hospital Shanghai University of Traditional Chinese Medicine, Shanghai, China

**Keywords:** diabetes mellitus, vascular endothelial growth factor inhibitors, chronic kidney disease, acute kidney injury, meta-analysis

## Abstract

**Background:**

Intravitreal vascular endothelial growth factor inhibitors (VEGFis) are a standard treatment for diabetic eye complications. However, concerns persist regarding their potential nephrotoxic effects in patients with diabetes mellitus (DM), who are inherently at increased risk of renal disease due to diabetes-related microvascular damage.

**Methods:**

We systematically searched PubMed, Embase, and Cochrane Library for randomized controlled trials (RCTs) evaluating renal-related adverse events in DM adults receiving intravitreal VEGFis *versus* controls. The primary outcome was occurrence of acute kidney injury (AKI), and the secondary outcome was the risk of chronic kidney disease (CKD). Fixed-effects models pooled odds ratios (ORs) with 95% confidence intervals (CIs).

**Results:**

From 16 RCTs (n = 5,930 patients), pooled analyses showed no significant increase in renal risk with VEGFis. The incidence of AKI (10 trials) showed no significant difference between the VEGFis groups (2.0%) and controls (1.5%; OR = 1.07, 95% CI: 0.65–1.75; GRADE very low quality). Similarly, the incidence of CKD (15 trials) was comparable in VEGFis groups (2.4%) *versus* controls (2.1%; OR = 1.11, 95% CI: 0.75–1.64; GRADE very low quality). Subgroup analyses of AKI incidence stratified by VEGFis types, injection numbers, and treatment duration showed similar event rates across all subgroups, with no statistically significant differences observed.

**Conclusion:**

Current evidence does not indicate a clear increase in the risk of AKI or CKD with intravitreal VEGFis in adults with DM, but the certainty is very low, and high-risk subgroups remain insufficiently studied.

## Graphical Abstract

**Figure:**
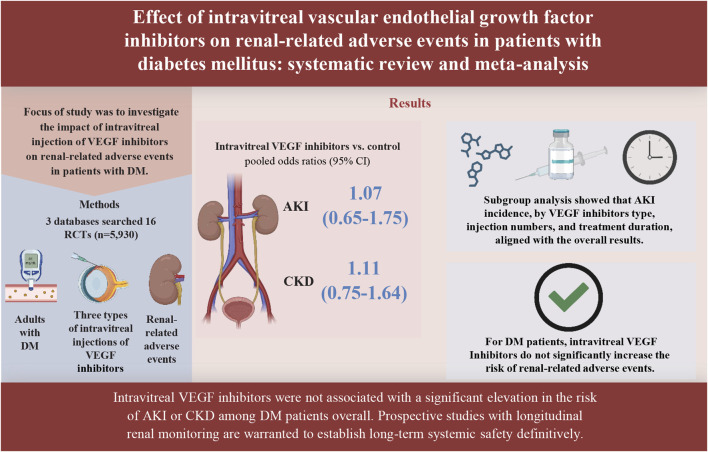
This figure was created with BioRender.com (https://www.biorender.com/).

## 1 Introduction

Diabetes mellitus (DM) represents a worldwide pandemic defined by sustained hyperglycemia and systemic microvascular sequelae; global prevalence in 2021 was no fewer than 529 million individuals (2023). Diabetic retinopathy (DR), recognized as the primary etiology of blindness in the working-age cohort (Sivaprasad et al., 2012), constitutes one of the most prevalent microvascular sequelae of DM (Teo et al., 2021). Diabetic macular edema (DME), a vision-threatening manifestation of DR (Hu et al., 2024), commonly requires therapy with vascular endothelial growth factor inhibitors (VEGFis) (Hanna et al., 2022) and significantly contributes to diabetes-related visual impairment. Intravitreal injections of VEGFis, including bevacizumab, ranibizumab, and aflibercept, have significantly improved the management of DR and DME (Hanna et al., 2022; Huang et al., 2025). Developed initially as systemic antiangiogenic agents for oncology, VEGFis were adapted for intravitreal ophthalmic applications in the early 2000s. Bevacizumab was initially administered off-label for retinal neovascularization, while aflibercept and ranibizumab subsequently received FDA approval for intravitreal use in 2007 and 2011, respectively (Hanna et al., 2022). Their efficacy in suppressing pathological angiogenesis and vascular permeability has been demonstrated to provide significant benefits in preserving and restoring visual acuity across multiple randomized controlled trials (RCTs) (Huang et al., 2025). In addition to ocular complications, DM frequently causes kidney disease. Diabetic kidney disease (DKD) affects approximately 40% of people with DM and is the leading cause of end-stage kidney disease (ESKD) worldwide; affected patients have an approximately threefold higher risk of all-cause mortality (Naaman and Bakris, 2023).

Although VEGFis are administered by intravitreal injection, systemic absorption has been documented, raising concerns regarding renal safety (Banerjee et al., 2025). Numerous studies have documented a decline in renal function following the administration of VEGFis (Ahmed et al., 2021; Zhang et al., 2021; Morales et al., 2017), with a significant number of cases involving the development of AKI (Zhang et al., 2021; Touzani et al., 2019; Hanna et al., 2020). The effects of VEGFis on renal function are particularly relevant in DM, given that pre-existing microvascular damage increases their susceptibility to renal disease (Huang et al., 2025). This concern arises from the role of VEGF in maintaining the glomerular filtration barrier. VEGF signaling supports the fenestrated glomerular endothelium and podocyte function, and its inhibition has been associated with loss of endothelial fenestrations and proteinuria (Hanna et al., 2020; Hanna et al., 2019a). Even a short drop in blood VEGF after an eye injection could disrupt key physiological functions (Banerjee et al., 2025).

Against this background, RCTs of intravitreal VEGFis mainly focus on ocular efficacy and safety, with limited data on their systemic effects, especially renal safety in patients with DM. While prior meta-analyses have explored renal-related adverse events (AEs), our study offers new insights by systematically evaluating a wide range of specific renal-related AEs in DM patients only (Huang et al., 2025; Lees et al., 2023). To address the lack of evidence on the renal effects of intravitreal VEGFis in DM, we conducted a systematic review and meta-analysis of RCTs.

This study aims to investigate the association between intravitreal VEGFis of bevacizumab, ranibizumab, or aflibercept and the risk of renal-related AEs, particularly AKI, in adult patients with DM.

## 2 Materials and methods

### 2.1 Registration

This systematic review and meta-analysis underwent pre-registration on the International Prospective Register of Systematic Reviews (PROSPERO; Registration ID: CRD 420251028391) and adhered to the 2020 PRISMA guidelines (Supplementary Table S1) (Page et al., 2021).

### 2.2 Data sources and search strategy

Employing predefined search protocols, PubMed, Embase, and Cochrane Library were systematically searched through integration of Medical Subject Headings (MeSH) and free-text keywords for: (1) diabetes mellitus, (2) bevacizumab, (3) ranibizumab, and (4) aflibercept. The search timeframe spanned from database inception through 2 March 2025, restricted to English-language publications. Full search strategies for individual databases are provided in Supplementary Table S2. Backward citation tracking of systematic reviews (Lees et al., 2023) was conducted to enhance literature identification. Retrieved records were managed using EndNote 20 (Clarivate) to ensure systematic organization.

### 2.3 Eligibility criteria

The PICOS framework (Population, Intervention, Comparator, Outcome, Study design) guided the definition of inclusion criteria: (1) Enrolled participants were adults (≥18 years) with clinically confirmed type 1/2 DM receiving intravitreal VEGFis therapy (bevacizumab, ranibizumab, or aflibercept), irrespective of ocular comorbidities or systemic conditions (e.g., cardiovascular/renal disorders). (2) Interventions were limited exclusively to protocol-specified VEGFis. (3) Comparator groups included sham injection controls or non-VEGFis therapies (e.g., laser photocoagulation). (4) The primary outcome was the occurrence of AKI, defined as either a 1.5-fold increase in serum creatinine level relative to baseline following intravitreal injection or an increase of ≥0.3 mg/dL within 48 h post-injection (Li et al., 2024). Secondary outcomes was CKD, defined as an estimated glomerular filtration rate (eGFR) < 60 mL/min/1.73 m^2^ or the presence of proteinuria (Liyanage et al., 2022). (5) Study design: Only RCTs with parallel-group designs were eligible for inclusion.

### 2.4 Selection process

A standardized duplicate independent review methodology was rigorously implemented throughout the selection process. Two independent investigators (YZ and FZ) performed initial screening of all identified records through parallel title/abstract evaluations, using the predefined PICOS framework as eligibility criteria. Full-text articles were retrieved when eligibility remained indeterminate based on title/abstract assessment alone. Resolution of reviewer discrepancies was achieved through either consensus-building or independent adjudication performed by a third party (XZ).

### 2.5 Data extraction

Two reviewers (YZ and FZ) independently performed duplicate data extraction for the following predefined domains: (1) bibliographic information (first author, publication year, country); (2) participant characteristics (mean age, DM classification); (3) Ophthalmic disease classification; (4) intervention specifications (VEGFis types, injection numbers, treatment duration, follow-up duration); (5) Outcome event counts for experimental and comparator arms. Disagreements regarding data interpretation underwent adjudication by a third investigator (XZ) to reach consensus.

To identify study eligibility, trial registration identifiers were cross-verified. When multiple reports existed for a single trial, the manuscript providing the longest follow-up with explicit documentation of relevant renal outcomes was prioritized for meta-analysis inclusion. For trials where baseline demographic profiles were incomplete in the primary report, supplementary data were extracted from the earliest associated publication.

### 2.6 Evaluation of study quality

Two independent reviewers (YZ and FZ) assessed the risk of bias for included RCTs using the Cochrane Revised Risk of Bias Tool-2 (RoB 2) (Sterne et al., 2019). This evaluation covered five core domains: (1) randomization process, (2) deviations from intended interventions, (3) missing outcome data, (4) outcome measurement, and (5) selection of reported results. All inter-reviewer variances underwent formal adjudication by a third author (XZ).

### 2.7 Evidence certainty assessment

We appraised evidence certainty for all outcomes using the GRADE framework via GRADEpro GDT (Brozek et al., 2009). Final ratings were determined by evaluating five core domains:

Risk of bias, indirectness, inconsistency, imprecision, and publication bias.

### 2.8 Data analysis

This study adopted a frequentist meta-analytic framework. Eligible studies that reported dichotomous outcomes were synthesized using the meta package in *R* software (version 4.3.2). Overall effects were evaluated using a z-test, with odds ratios (OR) and 95% confidence intervals (95% CI) as effect measures. To determine the probable range of the true effect in an individual setting, we calculated 95% prediction intervals (95% PIs) (IntHout et al., 2016). Heterogeneity was assessed using *I* ^*2*^ and τ^2^ (τ^2^ estimated by restricted maximum likelihood, REML), with *I* ^*2*^ ≥ 50% indicating substantial heterogeneity (Higgins et al., 2003). We prespecified a fixed-effects model when *I* ^*2*^ < 50% and a random-effects model otherwise. Continuity corrections of 0.5 were used to include zero total event trials (Yerubandi et al., 2024).

We also performed subgroup analyses of the primary outcome according to the VEGFis types, injection numbers, and treatment duration. Univariate meta-regression analyses were conducted on outcomes fulfilling the criterion of ≥10 studies per covariate (Ramirez-Campillo et al., 2022) to assess potential sources of heterogeneity, including VEGFis types, injection numbers, treatment duration, and follow-up duration. Sensitivity analysis was conducted using the leave-one-out approach. Publication bias was evaluated through visual assessment of funnel plots coupled with Egger’s regression test for asymmetry (West et al., 2010); *P* < 0.10 indicated statistical significance (Hanula et al., 2024; Hayashino et al., 2005).

## 3 Results

### 3.1 Characteristics of including RCTs

**FIGURE 1:**
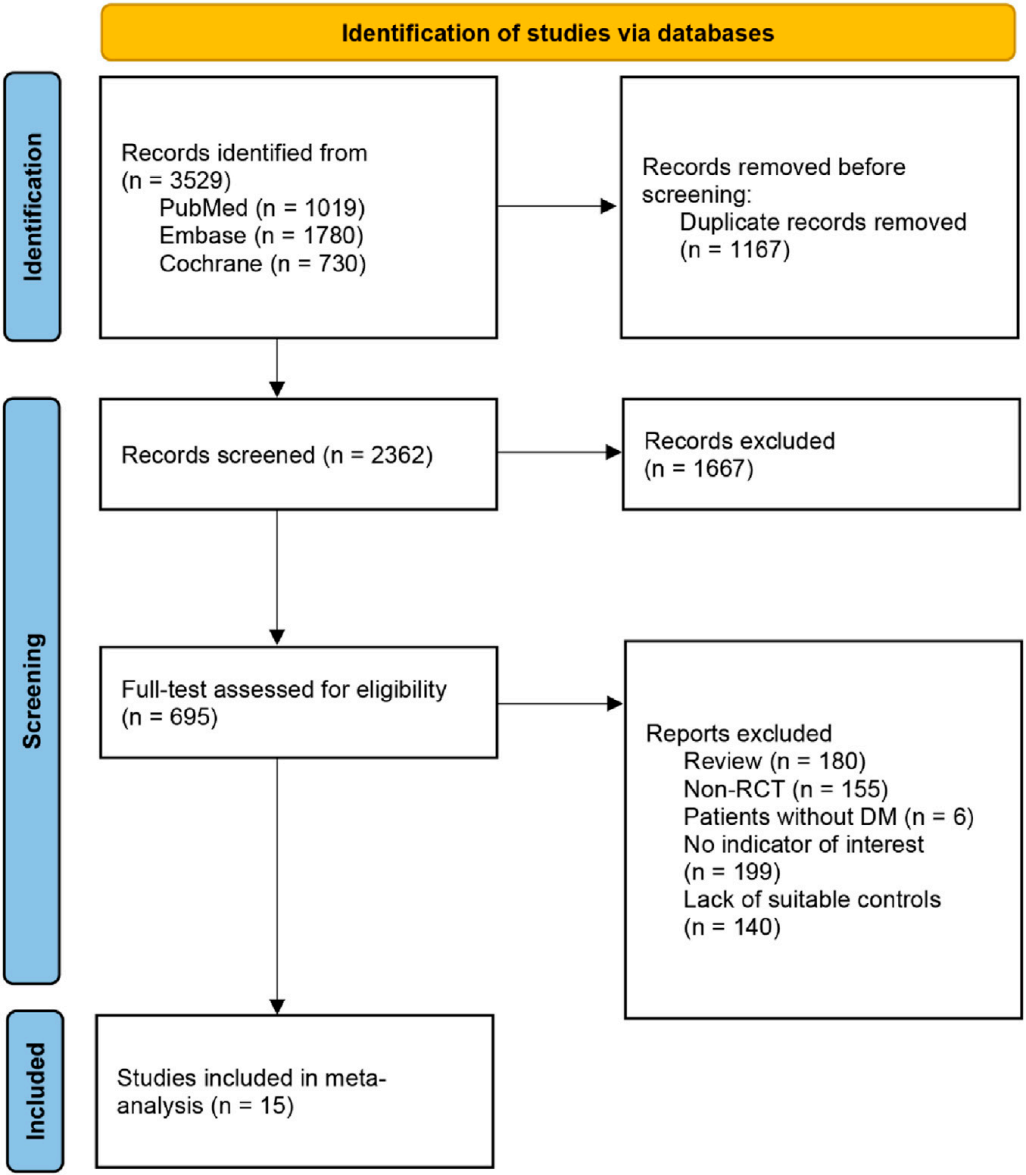
PRISMA flow diagram of the study selection process.

Through a systematic search of 3,529 articles, we identified 15 eligible full-text articles reporting 16 trials (Baker et al., 2019; Elman et al., 2010; Gale et al., 2018; Ishibashi et al., 2015; Li et al., 2019; Massin et al., 2010; Nguyen et al., 2012; Brown et al., 2021; Maturi et al., 2023; Gross et al., 2015; Petrarca et al., 2020; Mitchell et al., 2011; Brown et al., 2015; Gillies et al., 2014; Googe et al., 2011), with 5,930 participants (Figure 1). The mean age ranged from 52.0 to 63.9 years (Table 1). Ethnicity was recorded in 81.3% of studies. The evaluation of risk of bias across all included studies is presented in Supplementary Figure S1.

**TABLE 1:** Characteristic of included trials.

Author	Year	Trial ID	Country	Mean age (years)	Type of DM	Eye disease	Sample (n)	VEGFis types	Injections (n), mean	Treatment duration (months)	Follow-up duration (months)	Outcomes
(Baker et al., 2019)	2019	NCT01909791	USA	59.0	Type 1/2	DME	Int: 226 Con: 476	Aflibercept	8.3	24	24	CKD/AKI
(Elman et al., 2010)	2010	NCT00445003	USA	63.0	Type 1/2	DME	Int: 263 Con: 265 Bilateral: 163	Ranibizumab	9	12	24	CKD/AKI
(Gale et al., 2018)	2018	NCT01994291	International	62.3	Type 1/2	DME	Int: 99 Con: 99	Ranibizumab	3	3	4	CKD
(Ishibashi et al., 2015)	2015	NCT00989989	International	61.1	Type 1/2	DME	Int: 265 Con: 131	Ranibizumab	7	12	12	CKD
(Li et al., 2019)	2019	NCT02259088	China	58.7	Type 1/2	DME	Int: 307 Con: 77	Ranibizumab	7.9	12	12	CKD
(Massin et al., 2010)	2010	NCT00284050	France	63.6	Type 1/2	DME	Int: 102 Con: 49	Ranibizumab	10.2	12	12	CKD
(Nguyen et al., 2012)	2012	NCT00473330 (RISE)	USA	62.1	Type 1/2	DME	Int: 250 Con: 127	Ranibizumab	24	24	24	CKD/AKI
(Nguyen et al., 2012)	2012	NCT00473382 (RIDE)	USA	62.7	Type 1/2	DME	Int: 252 Con: 130	Ranibizumab	24	24	24	CKD/AKI
(Brown et al., 2021)	2021	NCT02718326	International	55.7	Type 1/2	NPDR	Int: 269 Con: 133	Aflibercept	9.1	23	23	AKI
(Maturi et al., 2023)	2023	NCT02634333	USA and Canada	56.1	Type 1/2	NPDR without CI-DME	Int: 129 Con: 128 Bilateral: 71	Aflibercept	13	48	48	CKD/AKI
(Gross et al., 2015)	2015	NCT01489189	USA	52.0	Type 1/2	PDR	Int: 102 Con: 114 Bilateral: 89	Ranibizumab	10.9	24	24	CKD/AKI
(Petrarca et al., 2020)	2019	NCT01030770	UK	63.9	Type 1/2	Persistent diabetic vitreous haemorrhage	Int: 12 Con: 12	Ranibizumab	1.1	12	12	CKD/AKI
(Mitchell et al., 2011)	2011	NCT00687804	International	63.5	Type 1/2	DME	Int: 234 Con: 111	Ranibizumab	6.9	12	12	CKD
(Brown et al., 2015)	2015	VISTA: NCT01363440 VIVID: NCT01331681	VISTA: USA VIVID: International	62.9	Type 1/2	DME	Int: 578 Con: 287	Aflibercept	17.8	22	23	CKD/AKI
(Gillies et al., 2014)	2014	NCT01298076	Australia	61.8	NR	DME	Int: 15 Con: 19 Bilateral: 27	Bevacizumab	8.6	12	12	CKD
(Googe et al., 2011)	2011	NCT00445003	USA	55.0	Type 1/2	DME	Int: 100 Con: 193 Bilateral: 26	Ranibizumab	2	3	13	CKD/AKI

DM: diabetes mellitus; DME: diabetic macular edema; PDR: proliferative diabetic retinopathy; CKD: chronic kidney disease; AKI: acute kidney injury; Int: Intervention; Con: Control; Bilateral: participants with two study eyes; NR: not reported.

There was one study of intravitreal bevacizumab, 11 studies of intravitreal ranibizumab, and four studies of intravitreal aflibercept [mean 10.2 injections (SD 6.8)]. The median treatment duration was 12 months (interquartile range [IQR] 12-24), and the median follow-up duration was 18 months (IQR 12-24) (Table 1).

### 3.2 Primary outcome

**FIGURE 2:**
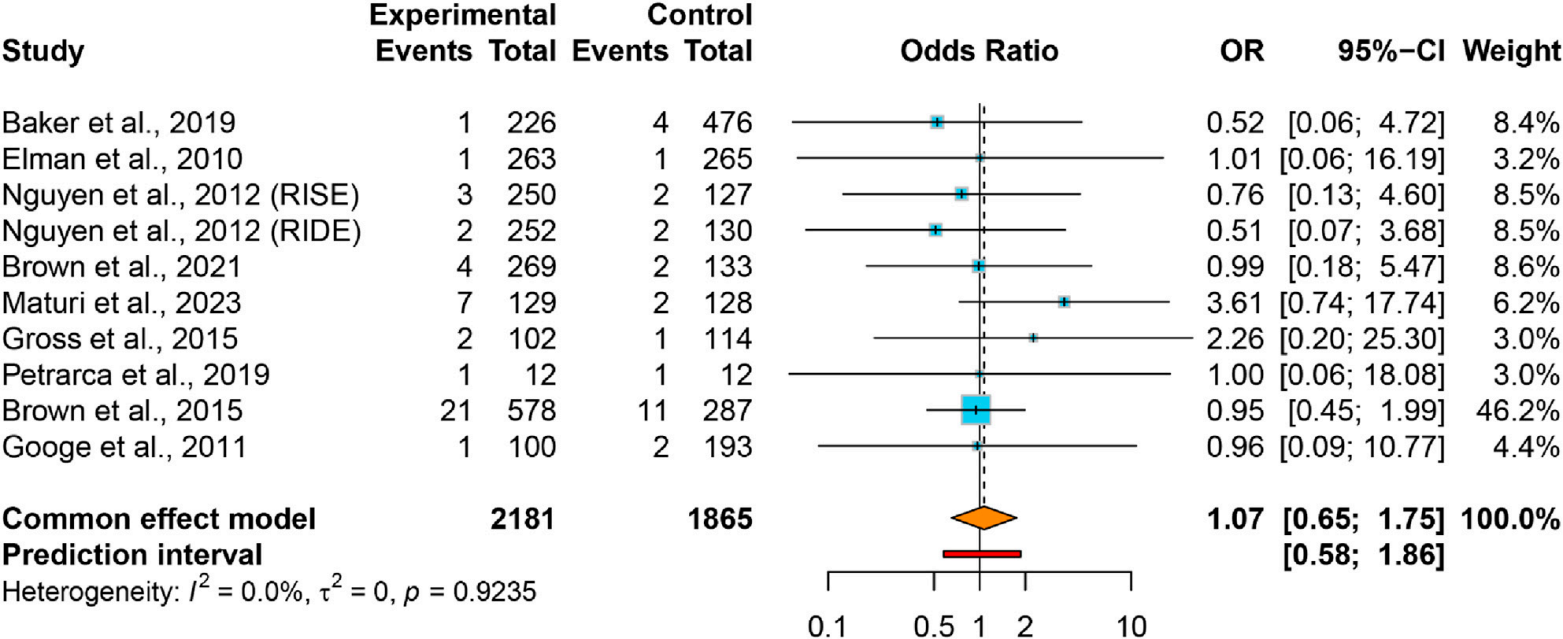
Forest plot of the ORs for AKI comparing VEGFis groups with control groups.

Pooled data revealed comparable AKI rates in VEGFis groups (2.0%) relative to controls (1.5%), yielding an OR of 1.07 (95% CI: 0.65–1.75) with negligible between-study heterogeneity (*I* ^*2*^ = 0.0%, τ^2^ = 0) (Figure 2). The 95% PI (0.58–1.86) indicates that future studies of intravitreal VEGFis injections are not expected to demonstrate a statistically significant increase in AKI incidence. Meta-regression analyses found no significant association between AKI risk and VEGFis types (*P* = 0.718), number of injections (*P* = 0.598), treatment duration (*P* = 0.187), or follow-up duration (*P* = 0.141); Supplementary Table S3). Sensitivity analyses confirmed result robustness (Supplementary Figure S2), while GRADE evaluation indicated very low evidence certainty (Table 2). Funnel plot symmetry and Egger’s test (*P* = 0.915) revealed negligible publication bias (Supplementary Figure S4).

**TABLE 2:** GRADE evidence profile for overall quality of evidence assessment.

Certainty assessment							No of patients		Effect		Certainty	Importance
No of studies	Study design	Risk of bias	Inconsistency	Indirectness	Imprecision	Other considerations	[VEGFis group]	[Control group]	Relative (95% CI)	Absolute (95% CI)		
AKI												
10	randomised trials	very serious^a^	not serious^b^	serious^c^	serious^d^	none	43/2181 (2.0%)	28/1865 (1.5%)	OR 1.07 (0.65–1.75)	0 fewer per 100 (from 0 fewer to 1 more)	⊕○○○ Very low^a^ ^,^ ^b^ ^,^ ^c^ ^,^ ^d^	Important
CKD												
15	randomised trials	very serious^a^	not serious^b^	serious^c^	serious^d^	none	71/2934 (2.4%)	47/2218 (2.1%)	OR 1.11 (0.75–1.64)	0 fewer per 100 (from 1 fewer to 1 more)	⊕○○○ Very low^a^ ^,^ ^b^ ^,^ ^c^ ^,^ ^d^	Important

CI: confidence interval; OR: odds ratio.

^a^ Potential high risk of bias in some trials

^b^ Low heterogeneity *I2* = < 50%

^c^ Outcomes of the RCTs were for the ophthalmic manifestations.

^d^ 95% CI crosses the line of null effect, with wide interval.

**FIGURE 3:**
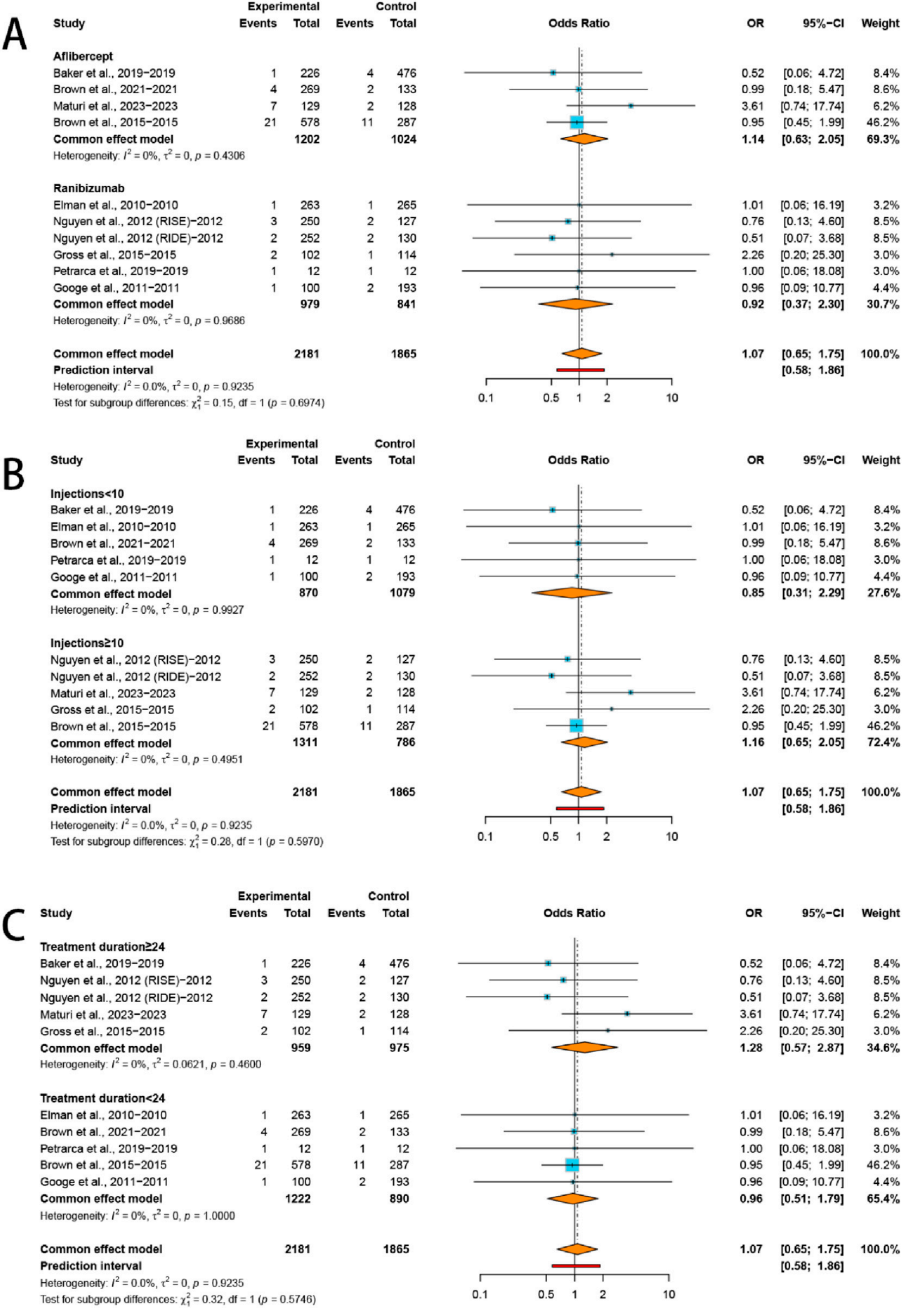
Subgroup analysis of the ORs for **(A)** VEGFis types, **(B)** injection numbers, and **(C)** treatment duration comparing VEGFis groups with control groups.

Our subgroup analysis revealed no statistically significant increase in the incidence of AKI within any of the predefined strata. Specifically, the risk of AKI was comparable between the aflibercept group (OR = 1.14, 95% CI: 0.63–2.05; *I* ^*2*^ = 0.0%) and the ranibizumab group (OR = 0.92, 95% CI: 0.37–2.30; *I* ^*2*^ = 0.0%). Similarly, no significant differences were observed across subgroups stratified by injections numbers (≥10 injections: OR = 1.16, 95% CI: 0.65–2.05, *I* ^*2*^ = 0.0%; <10 injections: OR = 0.85, 95% CI: 0.31–2.29, *I* ^*2*^ = 0.0%) or treatment duration (≥24 months: OR = 1.28, 95% CI: 0.57–2.87, *I* ^*2*^ = 0.0%; <24 months: OR = 0.96, 95% CI: 0.51–1.79, *I* ^*2*^ = 0.0%) (Figure 3).

### 3.3 Secondary outcome

**FIGURE 4:**
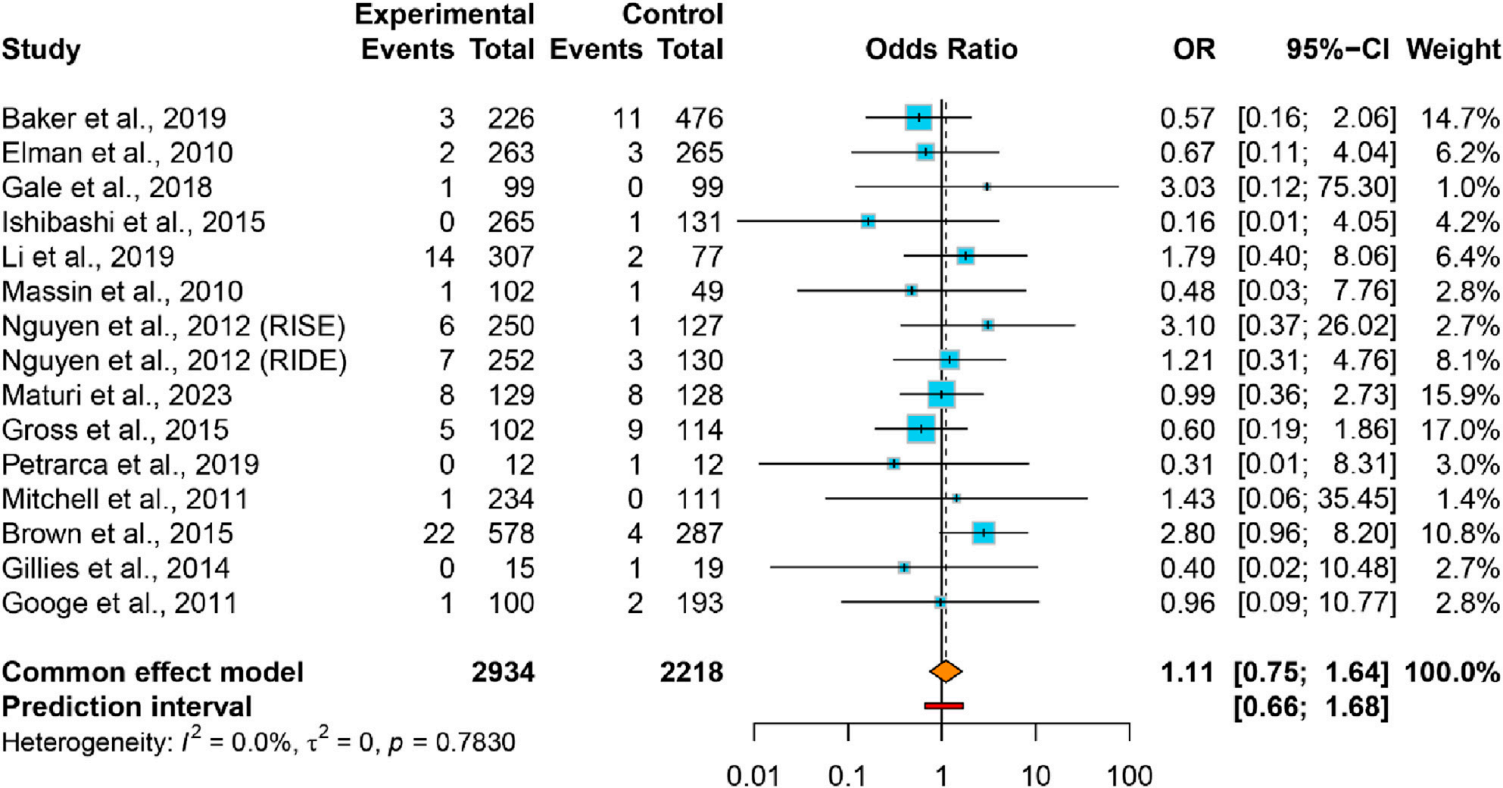
Forest plot of the ORs for CKD comparing VEGFis groups with control groups.

This meta-analysis encompassed 5,152 participants from 14 studies (15 trials) that documented incident CKD events. The CKD proportion in VEGFis groups (2.4%) demonstrated no significant elevation *versus* controls (2.1%) (OR = 1.11, 95% CI: 0.75–1.64), accompanied lower heterogeneity (*I* ^*2*^ = 0.0%, τ^2^ = 0) (Figure 4). The 95% PI (0.66–1.68) demonstrated no significant association between intravitreal VEGFis therapy and CKD incidence, with sensitivity analyses confirming result robustness (Supplementary Figure S3). Meta-regression analyses found no significant association between CKD risk and VEGFis types (*P* = 0.504; *P* = 0.547), injection numbers (*P* = 0.105), treatment duration (*P* = 0.994), or follow-up duration (*P* = 0.940; Supplementary Table S3). Evidence certainty was rated very low per GRADE criteria (Table 2). Funnel plot symmetry and Egger’s test (*P* = 0.402) indicated the absence of publication bias (Supplementary Figure S5).

## 4 Discussion

This meta-analysis of 16 RCTs (5,930 patients with DM) found no significant increase in AKI or CKD with intravitreal VEGFis *versus* controls, with low between-study heterogeneity. Subgroup analyses by agent (aflibercept vs. ranibizumab), injection numbers (≥10 vs. < 10), and treatment duration (≥24 vs. < 24 months) showed no difference in AKI. Meta-regression by agent, injection numbers, treatment duration, and follow-up likewise showed no association with renal risks. According to GRADE (Table 2), the certainty of evidence for AKI and CKD was very low due to risk of bias, indirectness, and imprecision; therefore, the results should be interpreted with caution.

DR progression is associated with incident DKD. Patients with advanced DR have about threefold higher DKD prevalence than those without DR (Orsi et al., 2023). Mechanistically, DR and DKD share hyperglycemia-driven microvascular pathways, including hyperfiltration, inflammation, and fibrosis (Alicic et al., 2017). In DR, disruption of the blood-retinal barrier driven by hyperglycemia with PKC activation, VEGF-A upregulation, and the plasma kallikrein-kinin system increases retinal vascular permeability (Zhang et al., 2014; Rask-Madsen and King, 2013). Together with documented systemic exposure after intravitreal anti-VEGF therapy, this provides a biologic basis for potential effects on renal VEGF-dependent pathways in podocytes and glomerular endothelium (Rask-Madsen and King, 2013; Avery et al., 2017). Case reports and a case series have reported worsening proteinuria, renal function decline, and hypertension after intravitreal VEGFis, indicating a potential safety signal (Ahmed et al., 2021; Hanna et al., 2019b).

In our meta-analysis of randomized trials in DM, intravitreal VEGFis and controls had similar risks of AKI and CKD. Although pooled estimates were slightly higher with VEGFis, the effects were not statistically significant. PIs further suggest that future studies, based on current evidence, are unlikely to demonstrate a significant overall increase in risk. A recent meta-analysis found no overall increase in cardiorenal events with intravitreal VEGFis; in diabetic eye disease, it noted higher all-cause mortality without a corresponding rise in kidney outcomes (Lees et al., 2023). These findings reinforce our conclusion that intravitreal anti-VEGF therapy in DM is not associated with an increased risk of renal AEs.

Consistent with these meta-analytic findings, an OHDSI multi-database cohort observed no difference in the risk of kidney failure among bevacizumab, ranibizumab, and aflibercept; the authors noted no empirical basis to prefer one agent for kidney protection and recommended monitoring kidney health during intravitreal anti-VEGF therapy (Cai et al., 2024). However, other real-world studies have reported associations with renal adverse outcomes. In a nationwide Veterans Health Administration cohort of adults with type 2 DM, patients receiving intravitreal anti-VEGF injections had a higher 5-year incidence of systemic AEs and higher adjusted odds of incident kidney disease (Zafar et al., 2023). A single-center matched cohort likewise showed faster eGFR decline and more dialysis after intravitreal VEGFis, especially in patients with pre-existing CKD (Rivero et al., 2024). In a Taiwanese cohort of patients with DME, aflibercept was associated with higher risks of composite renal AEs and AKI than ranibizumab; the associations were stronger in patients with pre-existing CKD and longer DM duration (Lee et al., 2025). Short-term clinical data are consistent: in a prospective study, patients with higher baseline urinary albumin-to-creatinine ratio had greater short-term increases in albuminuria after a single intravitreal bevacizumab injection (Chung et al., 2020). Taken together, these data suggest that any renal risk from intravitreal anti-VEGF therapy may be concentrated in DM patients with established DKD, rather than in the broader DM population. However, high-risk DKD populations are underrepresented in randomized trials, and renal endpoints were seldom prespecified or consistently reported, limiting inference for advanced CKD and reducing generalizability to higher-risk patients (Lees et al., 2023; Cai et al., 2024).

Systemic exposure after intravitreal dosing is influenced by molecular format. Because bevacizumab and aflibercept carry an Fc domain, they undergo FcRn-mediated recycling and reach higher and more prolonged systemic concentrations than the Fab fragment ranibizumab; accordingly, aflibercept and bevacizumab produce larger, and in some studies more persistent, reductions in circulating free VEGF (Avery et al., 2017; Jampol et al., 2018). On this pharmacologic background, a nationwide DME cohort from Taiwan reported higher incidence rates of adverse renal events with aflibercept than with ranibizumab (Lee et al., 2025). In contrast, our meta-regression and AKI subgroup analyses did not identify differences between drugs in renal AEs, consistent with an OHDSI network study showing similar kidney-failure risk across ranibizumab, aflibercept, and bevacizumab (Cai et al., 2024). Although pharmacokinetic and pharmacodynamic profiles differ across agents, comparative risk analyses have not established consistent between-drug differences in renal AEs, likely reflecting limitations in study design and statistical power (Zarbin, 2018).

For clinical implications, three points warrant emphasis. First, baseline renal assessment and monitoring during therapy are advised, especially in DKD. In patients with advanced DKD, baseline and periodic checks of serum creatinine and albuminuria, along with blood pressure monitoring, are advisable. Because renal status influences macular fluid dynamics, stabilizing kidney function may help sustain the response to intravitreal VEGFis in DME (Chou et al., 2024); prior studies show that worse renal function is associated with greater macular-edema fluctuation and higher peak central macular thickness (CMT), and that CMT improves after dialysis initiation (Usui-Ouchi et al., 2025). Second, risk communication. Pharmacovigilance shows no clear renal safety signal after intravitreal VEGFis, but the evidence base is limited, so long-term risk remains uncertain and should be discussed with patients (Jiang et al., 2023). Third, drug choice in high-risk patients. Given pharmacokinetic data, selecting an agent with lower systemic exposure (e.g., ranibizumab) may be reasonable, although comparative renal risk differences among agents have not been demonstrated (Jiang et al., 2023; Avery et al., 2014).

This study has several limitations. First, across all included RCTs, renal outcomes were safety endpoints rather than prespecified primary outcomes, and event counts were low, limiting robustness. In addition, short follow-up, with a median of 18 months across 16 RCTs, further constrained the assessment of CKD progression. Second, baseline renal status was poorly characterized in many trials, limiting subgroup analyses in high-risk patients; Protocol W (Maturi et al., 2023) noted prior kidney disease but did not tabulate creatinine, eGFR, or proteinuria and did not stratify outcomes by renal function; Gale (Gale et al., 2018) categorized baseline eGFR, stratified randomization by kidney function, and excluded eGFR <30 mL/min/1.73 m^2^ but did not report outcomes by eGFR strata; Googe (Googe et al., 2011) excluded substantial renal disease and did not provide baseline renal laboratory data. Third, definitions of renal outcomes varied. Only AKI and CKD were evaluated; other renal alterations, such as proteinuria and chronic glomerular injury, were not systematically ascertained and therefore cannot be excluded. No trial reported longitudinal eGFR, and renal data were generally captured as systemic AEs rather than repeated laboratory measurements.

Future studies should prioritize prospective real-world cohorts with larger samples, systematic renal monitoring, and extended follow-up to assess long-term systemic effects of VEGFis, particularly renal outcomes. Clinicians should periodically monitor renal parameters, especially in patients with pre-existing renal impairment or advanced DKD.

## 5 Conclusion

In adults with DM receiving intravitreal VEGFis, we did not find a clear increase in AKI or CKD compared with controls. However, the certainty of this evidence is very low; renal outcomes were secondary and infrequently reported, and high-risk subgroups remain insufficiently studied. These findings should be interpreted cautiously; prospective studies with systematic renal assessment are warranted.

## Data Availability

The original contributions presented in the study are included in the article/Supplementary Material, further inquiries can be directed to the corresponding authors.
