# Supplementary material for: Effect of intravitreal VEGF inhibitors on renal-related adverse events in patients with diabetes mellitus: systematic review and meta-analysis
Source: Front Pharmacol. 2025 Nov 14;16:1691597. doi: 10.3389/fphar.2025.1691597 (PMC12660107; doi:10.3389/fphar.2025.1691597)
Supplement: Supplementary file 1 [file Supplementaryfile1.docx]

**Table S1 The PRISMA 2020 Checklist**

| **Section and Topic** | **Item #** | **Checklist item** | **Location where item is reported** |
| --- | --- | --- | --- |
| **TITLE** | | |  |
| Title | 1 | Identify the report as a systematic review. | Title page |
| **ABSTRACT** | | |  |
| Abstract | 2 | See the PRISMA 2020 for Abstracts checklist. | Abstract |
| **INTRODUCTION** | | |  |
| Rationale | 3 | Describe the rationale for the review in the context of existing knowledge. | 1. Introduction |
| Objectives | 4 | Provide an explicit statement of the objective(s) or question(s) the review addresses. | 1. Introduction |
| **METHODS** | | |  |
| Eligibility criteria | 5 | Specify the inclusion and exclusion criteria for the review and how studies were grouped for the syntheses. | 2.3 Eligibility criteria |
| Information sources | 6 | Specify all databases, registers, websites, organisations, reference lists and other sources searched or consulted to identify studies. Specify the date when each source was last searched or consulted. | 2.2 Data sources and search strategy |
| Search strategy | 7 | Present the full search strategies for all databases, registers and websites, including any filters and limits used. | Supplementary Table S2 |
| Selection process | 8 | Specify the methods used to decide whether a study met the inclusion criteria of the review, including how many reviewers screened each record and each report retrieved, whether they worked independently, and if applicable, details of automation tools used in the process. | 2.4 Selection process |
| Data collection process | 9 | Specify the methods used to collect data from reports, including how many reviewers collected data from each report, whether they worked independently, any processes for obtaining or confirming data from study investigators, and if applicable, details of automation tools used in the process. | 2.5 Data extraction |
| Data items | 10a | List and define all outcomes for which data were sought. Specify whether all results that were compatible with each outcome domain in each study were sought (e.g. for all measures, time points, analyses), and if not, the methods used to decide which results to collect. | 2.3 Eligibility criteria |
|  | 10b | List and define all other variables for which data were sought (e.g. participant and intervention characteristics, funding sources). Describe any assumptions made about any missing or unclear information. | 2.5 Data extraction |
| Study risk of bias assessment | 11 | Specify the methods used to assess risk of bias in the included studies, including details of the tool(s) used, how many reviewers assessed each study and whether they worked independently, and if applicable, details of automation tools used in the process. | 2.6 Evaluation of study quality |
| Effect measures | 12 | Specify for each outcome the effect measure(s) (e.g. risk ratio, mean difference) used in the synthesis or presentation of results. | 2.8 Data analysis |
| Synthesis methods | 13a | Describe the processes used to decide which studies were eligible for each synthesis (e.g. tabulating the study intervention characteristics and comparing against the planned groups for each synthesis (item #5)). | 2.8 Data analysis |
|  | 13b | Describe any methods required to prepare the data for presentation or synthesis, such as handling of missing summary statistics, or data conversions. | 2.8 Data analysis |
|  | 13c | Describe any methods used to tabulate or visually display results of individual studies and syntheses. | 2.8 Data analysis |
|  | 13d | Describe any methods used to synthesize results and provide a rationale for the choice(s). If meta-analysis was performed, describe the model(s), method(s) to identify the presence and extent of statistical heterogeneity, and software package(s) used. | 2.8 Data analysis |
|  | 13e | Describe any methods used to explore possible causes of heterogeneity among study results (e.g. subgroup analysis, meta-regression). | 2.8 Data analysis |
|  | 13f | Describe any sensitivity analyses conducted to assess robustness of the synthesized results. | 2.8 Data analysis |
| Reporting bias assessment | 14 | Describe any methods used to assess risk of bias due to missing results in a synthesis (arising from reporting biases). | 2.8 Data analysis |
| Certainty assessment | 15 | Describe any methods used to assess certainty (or confidence) in the body of evidence for an outcome. | 2.7 Evidence certainty assessment |
| **RESULTS** | | |  |
| Study selection | 16a | Describe the results of the search and selection process, from the number of records identified in the search to the number of studies included in the review, ideally using a flow diagram. | Figure 1 |
|  | 16b | Cite studies that might appear to meet the inclusion criteria, but which were excluded, and explain why they were excluded. | Figure 1 |
| Study characteristics | 17 | Cite each included study and present its characteristics. | Table 1 |
| Risk of bias in studies | 18 | Present assessments of risk of bias for each included study. | Supplementary Figure S1 |
| Results of individual studies | 19 | For all outcomes, present, for each study: (a) summary statistics for each group (where appropriate) and (b) an effect estimate and its precision (e.g. confidence/credible interval), ideally using structured tables or plots. | Figure 2 |
| Results of syntheses | 20a | For each synthesis, briefly summarise the characteristics and risk of bias among contributing studies. | 3.2 Primary outcome; 3.3 Secondary outcomes |
|  | 20b | Present results of all statistical syntheses conducted. If meta-analysis was done, present for each the summary estimate and its precision (e.g. confidence/credible interval) and measures of statistical heterogeneity. If comparing groups, describe the direction of the effect. | 3.2 Primary outcome; 3.3 Secondary outcomes |
|  | 20c | Present results of all investigations of possible causes of heterogeneity among study results. | 3.2 Primary outcome; 3.3 Secondary outcomes; Table S3 |
|  | 20d | Present results of all sensitivity analyses conducted to assess the robustness of the synthesized results. | Supplementary Figure S2, S3 |
| Reporting biases | 21 | Present assessments of risk of bias due to missing results (arising from reporting biases) for each synthesis assessed. | Supplementary Figure S4, S5 |
| Certainty of evidence | 22 | Present assessments of certainty (or confidence) in the body of evidence for each outcome assessed. | Table 2 |
| **DISCUSSION** | | |  |
| Discussion | 23a | Provide a general interpretation of the results in the context of other evidence. | 4. Discussion |
|  | 23b | Discuss any limitations of the evidence included in the review. | 4. Discussion |
|  | 23c | Discuss any limitations of the review processes used. | 4. Discussion |
|  | 23d | Discuss implications of the results for practice, policy, and future research. | 4. Discussion |
| **OTHER INFORMATION** | | |  |
| Registration and protocol | 24a | Provide registration information for the review, including register name and registration number, or state that the review was not registered. | 2.1 Registration |
|  | 24b | Indicate where the review protocol can be accessed, or state that a protocol was not prepared. | 2.1 Registration |
|  | 24c | Describe and explain any amendments to information provided at registration or in the protocol. | 2.1 Registration |
| Support | 25 | Describe sources of financial or non-financial support for the review, and the role of the funders or sponsors in the review. | Funding |
| Competing interests | 26 | Declare any competing interests of review authors. | Declaration of competing interest |
| Availability of data, code and other materials | 27 | Report which of the following are publicly available and where they can be found: template data collection forms; data extracted from included studies; data used for all analyses; analytic code; any other materials used in the review. | Data availability statement |

**Table S2 Search detailed for database**

| PubMed | | |  |
| --- | --- | --- | --- |
| NO. | Elements | Search detail |  |
| 1 | Population | (Diabetes Mellitus[MeSH Terms]) OR (Diabetes Mellitus[Title/Abstract] OR Diabetes[Title/Abstract] OR Diabetic[Title/Abstract] OR type 2 diabetes[Title/Abstract] OR T2DM[Title/Abstract] OR Diabet*[Title/Abstract] OR type 1 diabetes[Title/Abstract] OR T1DM[Title/Abstract] OR diabetes mellitus, type 1[Title/Abstract] OR diabetes mellitus, type 2[Title/Abstract]) | 923341 |
| 2 | Intervention | (Intravitreal Injections[MeSH Terms]) OR (Intravitreal Injections[Title/Abstract] OR Injection, Intravitreal[Title/Abstract] OR Injections, Intravitreal[Title/Abstract] OR Intravitreal Injection[Title/Abstract]) | 18293 |
| 3 | Intervention | (((((Bevacizumab[MeSH Terms]) OR (Ranibizumab[MeSH Terms])) OR (Aflibercept[Supplementary Concept])) OR (Bevacizumab[Title/Abstract] OR Avastin[Title/Abstract] OR Mvasi[Title/Abstract] OR Bevacizumab-awwb[Title/Abstract] OR Bevacizumab awwb[Title/Abstract])) OR (Ranibizumab[Title/Abstract] OR RhuFab V2[Title/Abstract] OR V2, RhuFab[Title/Abstract] OR Lucentis[Title/Abstract])) OR (Aflibercept[Title/Abstract] OR VEGF-Trap[Title/Abstract] OR VEGF Trap – Regeneron[Title/Abstract] OR VEGF Trap-Eye[Title/Abstract] OR AVE 005[Title/Abstract] OR AVE-005[Title/Abstract] OR AVE005[Title/Abstract] OR AVE 0005[Title/Abstract] OR AVE-0005[Title/Abstract] OR AVE0005[Title/Abstract] OR eylea[Title/Abstract] OR ZIV-aflibercept[Title/Abstract] OR Zaltrap[Title/Abstract]) | 31567 |
| 4 | Study design | (Randomized Controlled Trial[Publication Type]) OR (Randomized Controlled Trial[Title/Abstract] OR controlled clinical trial[Title/Abstract] OR Clinical Trial[Title/Abstract] OR randomized[Title/Abstract] OR random[Title/Abstract] OR randomly[Title/Abstract] OR randomised[Title/Abstract] OR placebo[Title/Abstract] OR trial[Title/Abstract] OR groups[Title/Abstract]) |  |
| 5 |  | #1 AND #2 AND #3 AND #4 | 1019 |
| Embase | | |  |
| NO. | Elements | Search detail |  |
| 1 | Population | 'diabetes mellitus'/exp OR 'diabetes mellitus' OR 'diabetes'/exp OR 'diabetes' OR 'diabetic'/exp OR 'diabetic' OR 'type 2 diabetes'/exp OR 'type 2 diabetes' OR 't2dm'/exp OR 't2dm' OR 'diabet*' OR 'type 1 diabetes'/exp OR 'type 1 diabetes' OR 't1dm'/exp OR 't1dm' OR 'diabetes mellitus, type 1'/exp OR 'diabetes mellitus, type 1' OR 'diabetes mellitus, type 2'/exp OR 'diabetes mellitus, type 2' | 1825105 |
| 2 | Intervention | 'intravitreal injections'/exp OR 'intravitreal injections' OR 'injection, intravitreal' OR 'injections, intravitreal' OR 'intravitreal injection' | 25428 |
| 3 | Intervention | 'bevacizumab'/exp OR 'bevacizumab' OR 'avastin'/exp OR 'avastin' OR 'mvasi'/exp OR 'mvasi' OR 'bevacizumab-awwb'/exp OR 'bevacizumab-awwb' OR 'bevacizumab awwb'/exp OR 'bevacizumab awwb' OR 'ranibizumab'/exp OR 'ranibizumab' OR 'rhufab v2'/exp OR 'rhufab v2' OR 'v2, rhufab' OR 'lucentis'/exp OR 'lucentis' OR 'aflibercept'/exp OR 'aflibercept' OR 'vegf-trap'/exp OR 'vegf-trap' OR 'vegf trap – regeneron' OR 'vegf trap-eye'/exp OR 'vegf trap-eye' OR 'ave 005'/exp OR 'ave 005' OR 'ave-005'/exp OR 'ave-005' OR 'ave005'/exp OR 'ave005' OR 'ave 0005'/exp OR 'ave 0005' OR 'ave-0005'/exp OR 'ave-0005' OR 'ave0005'/exp OR 'ave0005' OR 'eylea'/exp OR 'eylea' OR 'ziv-aflibercept'/exp OR 'ziv-aflibercept' OR 'zaltrap'/exp OR 'zaltrap' | 95756 |
| 4 | Study design | 'randomized controlled trial'/exp OR 'randomized controlled trial' OR 'controlled clinical trial'/exp OR 'controlled clinical trial' OR 'clinical trial'/exp OR 'clinical trial' OR 'randomized' OR 'random' OR 'randomly' OR 'randomised' OR 'placebo'/exp OR 'placebo' OR 'trial'/exp OR 'trial' OR 'groups' | 7231655 |
| 5 |  | #1 AND #2 AND #3 AND #4 | 1780 |
| Cochrane | | |  |
| NO. | Elements | Search detail |  |
| 1 | Population | MeSH descriptor: [Diabetes Mellitus] explode all trees | 46179 |
| 2 | Population | ("Diabetes Mellitus" OR Diabetes OR Diabetic OR "type 2 diabetes" OR T2DM OR Diabet* OR "type 1 diabetes" OR T1DM OR "diabetes mellitus, type 1" OR "diabetes mellitus, type 2"):ti,ab,kw (Word variations have been searched) | 126383 |
| 3 | Population | #1 OR #2 | 126760 |
| 4 | Intervention | MeSH descriptor: [Intravitreal Injections] explode all trees | 1357 |
| 5 | Intervention | ("Intravitreal Injections" OR "Injection, Intravitreal" OR "Injections, Intravitreal" OR "Intravitreal Injection"):ti,ab,kw (Word variations have been searched) | 2879 |
| 6 | Intervention | #4 OR #5 | 2879 |
| 7 | Intervention | MeSH descriptor: [Bevacizumab] explode all trees | 3036 |
| 8 | Intervention | (Bevacizumab OR Avastin OR Mvasi OR "Bevacizumab-awwb" OR "Bevacizumab awwb"):ti,ab,kw (Word variations have been searched) | 8159 |
| 9 | Intervention | #7 OR #8 | 8159 |
| 10 | Intervention | MeSH descriptor: [Ranibizumab] explode all trees | 1191 |
| 11 | Intervention | (Ranibizumab OR "RhuFab V2" OR "V2, RhuFab" OR "Lucentis"):ti,ab,kw (Word variations have been searched) | 2350 |
| 12 | Intervention | #10 OR #11 | 2350 |
| 13 | Intervention | (Aflibercept OR VEGF-Trap OR "VEGF Trap-Regeneron" OR "VEGF Trap-Eye" OR AVE 005 OR AVE-005 OR AVE005 OR AVE 0005 OR AVE-0005 OR AVE0005 OR eylea OR ZIV-aflibercept OR Zaltrap):ti,ab,kw (Word variations have been searched) | 1367 |
| 14 | Intervention | #9 OR #12 OR #13 | 10905 |
| 15 | Study design | MeSH descriptor: [Randomized Controlled Trial] explode all trees | 34 |
| 16 | Study design | ("Randomized Controlled Trial" OR "controlled clinical trial" OR "Clinical Trial" OR randomized OR random OR randomly OR randomised OR placebo OR trial OR groups):ti,ab,kw (Word variations have been searched) | 1653111 |
| 17 | Study design | #15 OR #16 | 1653111 |
| 18 |  | #3 AND #6 AND #14 AND #17 | 730 |

**Table S3 Meta-regression models to assess potential sources of heterogeneity for rate of each outcome**

| **AKI** |  | **Coefficient** | **95% CI** | ***P* value** | **Tau^2^** | ***I^2^*** |
| --- | --- | --- | --- | --- | --- | --- |
| VEGFis type | Ranibizumab | -0.2038 | -1.3117, 0.9040 | 0.718 | 0 | 0.00% |
| Number of injections |  | -0.0226 | -0.1064, 0.0613 | 0.598 | 0 | 0.00% |
| Treatment duration |  | 0.0362 | -0.0175, 0.0899 | 0.187 | 0 | 0.00% |
| Follow-up duration |  | 0.0463 | -0.0153, 0.1079 | 0.141 | 0 | 0.00% |
| **CKD** |  | **Coefficient** | **95% CI** | ***P* value** | **Tau^2^** | ***I^2^*** |
| VEGFis type | Bevacizumab | -1.1407 | -4.4866, 2.2053 | 0.504 | 0.0169 | 2.18% |
|  | Ranibizumab | -0.2705 | -1.1519, 0.6109 | 0.547 |  |  |
| Number of injections |  | 0.0588 | -0.0124, 0.1300 | 0.105 | 0 | 0.00% |
| Treatment duration |  | -0.0001 | -0.0362, 0.0359 | 0.994 | 0.0459 | 5.38% |
| Follow-up duration |  | -0.0015 | -0.0403, 0.0373 | 0.940 | 0.0457 | 5.33% |
| CKD: chronic kidney disease; AKI: acute kidney injury; VEGFis: vascular endothelial growth factor inhibitors. | | | | | | |

**Figure S1 Assessment of bias for inclusion in randomized controlled trials**
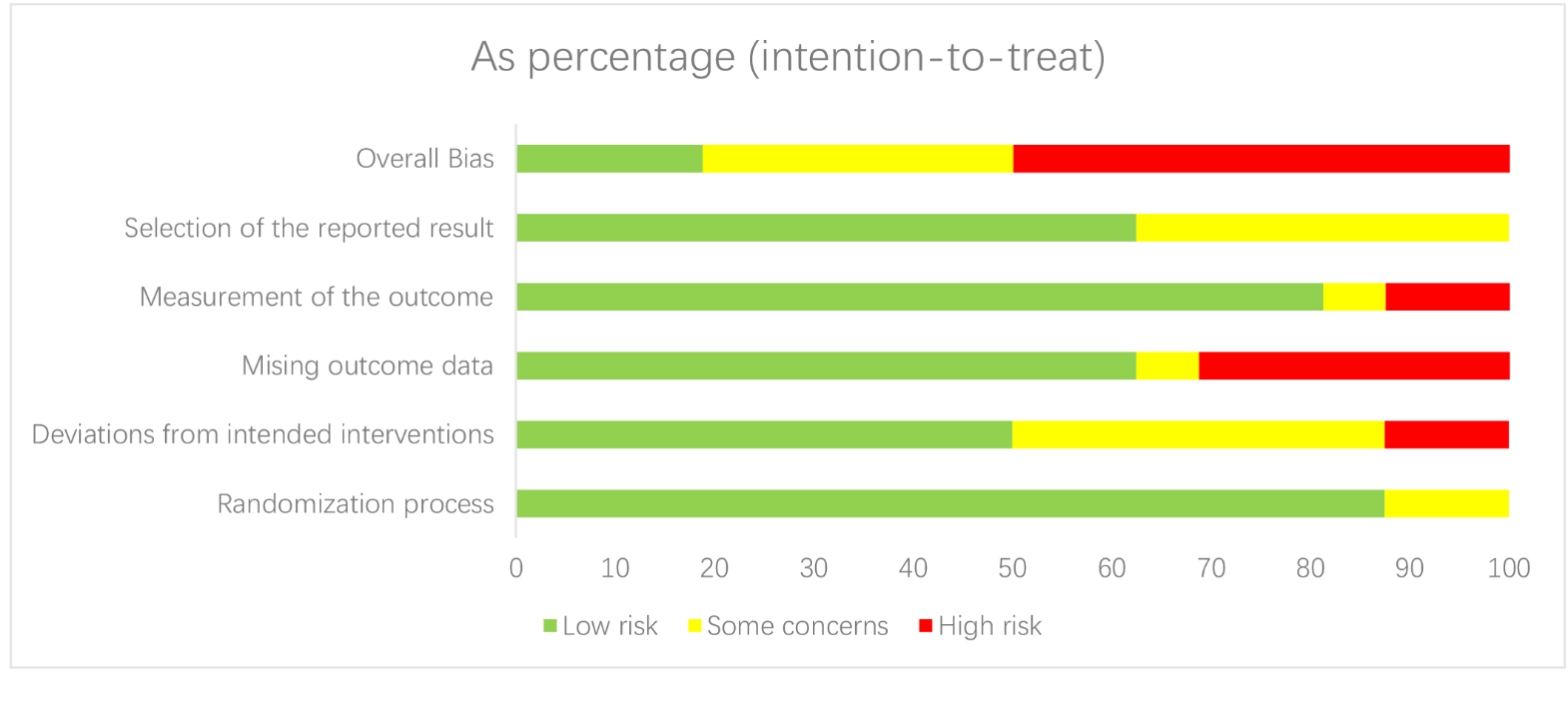


**Figure S2 Sensitivity analysis of AKI**





Note: Leave-one-out sensitivity analysis for meta-analysis; OR, odds ratio; 95% CI, 95% confidence interval.

**Figure S3 Sensitivity analysis of CKD**





Note: Leave-one-out sensitivity analysis for meta-analysis; OR, odds ratio; 95% CI, 95% confidence interval.

**Figure S4 Funnel plot assessing publication bias for AKI**





**Figure S5 Funnel plot assessing publication bias for CKD**

**
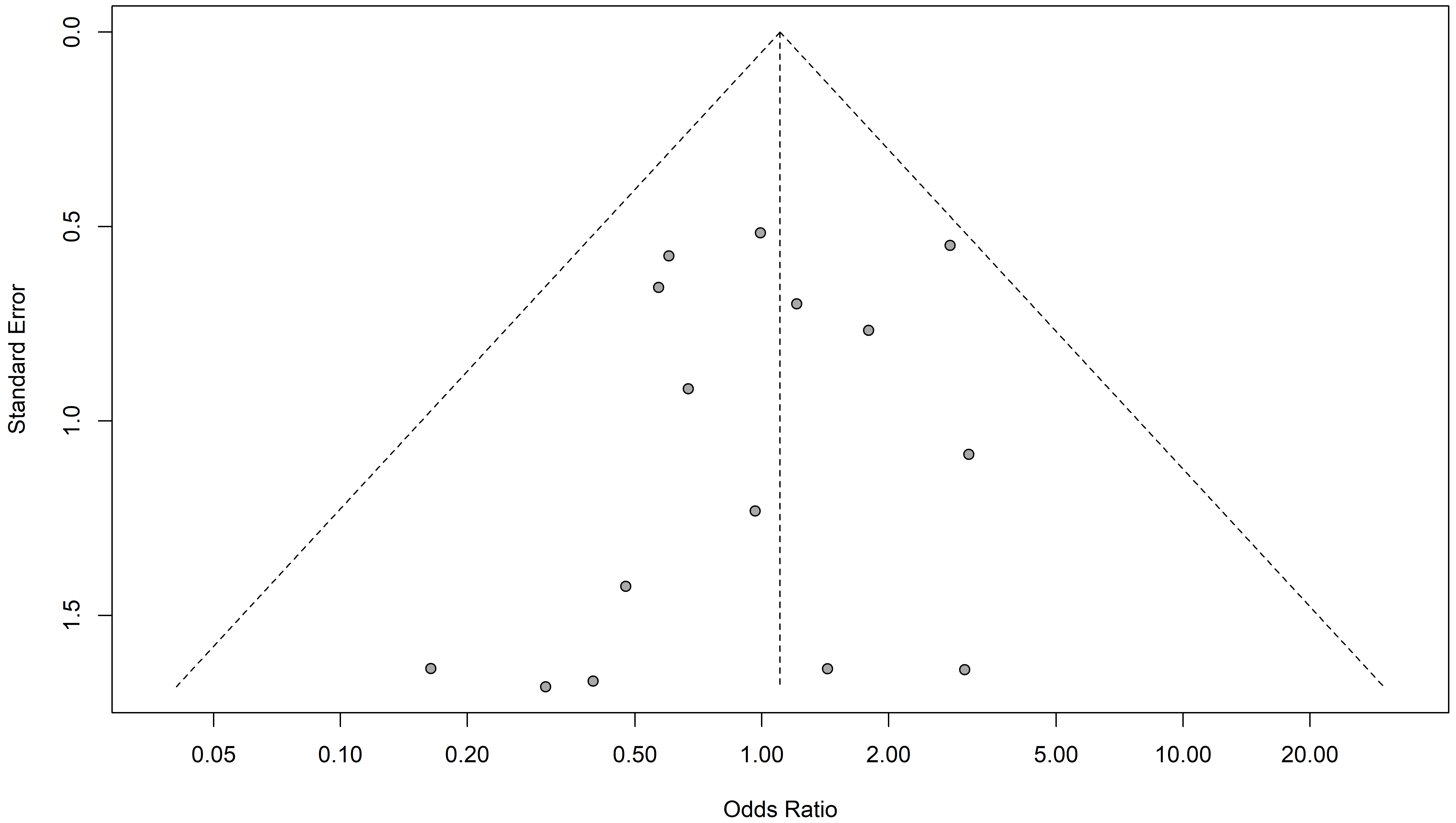
**
